# Mechanism and regioselectivity of electrophilic aromatic nitration in solution: the validity of the transition state approach

**DOI:** 10.1007/s00894-017-3561-z

**Published:** 2017-12-18

**Authors:** Magnus Liljenberg, Joakim Halldin Stenlid, Tore Brinck

**Affiliations:** Applied Physical Chemistry, KTH Royal Institute of Technology, S-100 44 Stockholm, Sweden

**Keywords:** Nitration, Electrophilic aromatic substitution, Transition state, Regioselectivity, Quantum chemistry

## Abstract

The potential energy surfaces in gas phase and in aqueous solution for the nitration of benzene, chlorobenzene, and phenol have been elucidated with density functional theory at the M06-2X/6-311G(d,p) level combined with the polarizable continuum solvent model (PCM). Three reaction intermediates have been identified along both surfaces: the unoriented π-complex (I), the oriented reaction complex (II), and the σ-complex (III). In order to obtain quantitatively reliable results for positional selectivity and for modeling the expulsion of the proton, it is crucial to take solvent effects into consideration. The results are in agreement with Olah’s conclusion from over 40 years ago that the transition state leading to (II) is the rate-determining step in activated cases, while it is the one leading to (III) for deactivated cases. The simplified reactivity approach of using the free energy for the formation of (III) as a model of the rate-determining transition state has previously been shown to be very successful for halogenations, but problematic for nitrations. These observations are rationalized with the geometric and energetic resemblance, and lack of resemblance respectively, between (III) and the corresponding rate determining transition state. At this level of theory, neither the σ-complex (III) nor the reaction complex (II) can be used to accurately model the rate-determining transition state for nitrations.

## Introduction

**Fig. 1:**
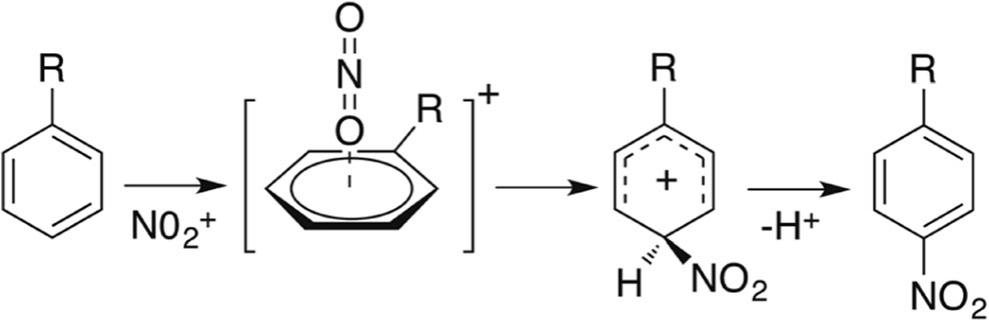
The putative mechanism for S_E_Ar nitrations

Electrophilic aromatic nitration is one of the most thoroughly studied classes of organic reactions, and its mechanism has been intensely debated over many decades [1–7]. Still it continues to fascinate. The active electrophile for nitration is widely believed to be the nitronium ion (NO_2_^+^), and the putative mechanism for the generic reaction is outlined in Fig. 1 [8]. The first step is the, usually rapid and reversible, complexation of NO_2_^+^ with the π-system of the aromatic ring, a species commonly referred to as the π-complex. This species is unoriented, that is no positional selectivity is associated with it. In order for the substitution process to proceed, the π-complex must react to form another reaction intermediate, the σ-complex, a species that is also known as the Wheland intermediate or the arenium ion. In this intermediate the cyclic conjugation of the aromatic system is broken, and the carbon at the site of substitution is tetravalent and bonded via σ-bonds to both the nitrogen of the NO_2_^+^ electrophile as well as to the leaving group (H^+^). For nitrations, the formation of the σ-complex is essentially irreversible and in the last step a proton is eliminated, giving the product. The formation of the σ-complex is almost always the rate-limiting step [8, 9]. Melander first recognized this by showing that nitrations in general lack an isotope effect [1].

Many computational studies have been devoted to the elucidation of the mechanism of this classical reaction [5–7, 10–13]. Most of these have focused on gas phase conditions. One of the first studies to analyze the potential energy surface for nitration using ab initio quantum chemistry was conducted by Politzer and coworkers [10]. Their study involved both benzene and toluene and indicated the presence of an oriented reaction complex (II) that precedes the formation of the classical σ-complex (III). Esteves et al. [5] later calculated the detailed potential energy surface of the reaction between benzene and NO_2_^+^ in gas phase by restricted Kohn-Sham density functional theory (KS-DFT) with the B3LYP functional. They identified three key intermediates: the first (I) is an unoriented π-complex of C_6v_ symmetry with a perpendicular coordination of the NO_2_ group toward the center of the aromatic ring. The second (II) is an oriented reaction complex, which Esteves et al. called “a cation radical molecule intermediate pair” with the nitrogen of the NO_2_ group positioned above the respective ring carbon. The third (III) is the σ-complex. According to the authors, these three intermediates unify previous mechanistic proposals. In a second study Esteves and coworkers investigated the nitration (with both the naked NO_2_^+^ and the monosolvated CH_3_NO_2_^•^NO_2_ nitronium ion as electrophiles) of a number of monosubstituted benzenes in gas phase [12]. They suggest that the attack of the nitronium ion follows a mechanistic continuum with a single-electron transfer mechanism (SET) and a polar (Ingold-Hughes) mechanism as the two extreme endpoints. Activating substituents and aprotic polar solvents favor the SET mechanism, whereas deactivating substituents and protic polar solvents favor the Ingold-Hughes mechanism. The second C-atom coordinated π-complex (II) was, in line with their earlier study, considered to be a radical-radical cation complex formed from the SET.

Gwaltney et al. [11] investigated the mechanism for both nitration (NO_2_^+^) and nitrosation (NO^+^) of benzene in gas phase using coupled cluster theory (CCSD(T)/6-31G(d,p)). The intermediates identified in their analysis correspond to the reaction complex (II) and the σ-complex (III). They found that the nitrosonium reaction is different from nitrations in that it features no stable σ-complex; the bonded σ-complex (Wheland structure) is rather a transition state (TS) of the reaction. The authors advocate that the gas phase reaction proceeds via a SET-mechanism, but with the significant difference from Esteves et al. that the electron transfer takes place upon the formation of the σ-complex.

Xu et al. [13] investigated the reaction between NO_2_^+^ and benzene in the gas phase using multiconfigurational SCF theory at the CAS-SCF(10,9)/cc-*p*VDZ level. They found that the interacting system is on the ground state at all investigated distances, even though the separated reactants are in an excited state. This is attributed to the small difference in ionization potential between NO_2_ and benzene, and the large reorganization energy in the reduction of NO_2_^+^. The reaction proceeds via a C-coordinated π-complex (II) that converts to the sigma-complex via a small barrier. The authors conclude that a SET-mechanism is favored for aromatics that are more activated than benzene. They also state that SET is not likely to play a role in solution.

There have also been many computational studies focusing on the positional selectivity in nitrations of arenes. The most direct approach for computing the regioselectivity is to characterize all the stationary points along the PES and to estimate the rate constants from the free energy difference between the rate-determining transition states. A number of studies have used this approach, without fully reproducing the observed isomer distribution [7, 14–16]. An approximate variant is to use the energy of the corresponding σ-complex structure as an indicator of the transition state energy (the “σ-complex approach”). In a recent article we have reported on the use of the σ-complex approach for predicting positional selectivity for halogenations, nitrations, and Friedel-Crafts acylations in solution [17]. We found that it provides quantitative accuracy for halogenations, but that it fails for the nitration of monosubstituted benzenes. More specifically, the method predicts a too high energy barrier for the formation of the ortho isomer relative to the para and meta isomers for all investigated monosubstituted benzenes, with the result that the amount of the ortho isomer is consistently underestimated. The σ-complex approach also predicts a too high relative barrier for the meta isomer in the cases of ortho/para directing substituents. Whereas the σ-complex approach is not suitable to predict the positional selectivity of nitrations, it can provide a quantitative measure of the global reactivity. Galabov and coworkers reported good correlations between relative reaction rates and the σ-complex stabilization energy for the nitration of monosubstituted benzenes as well as for halogenations and alkylations (benzylation) [18, 19].

In a very recent study Nieves-Quinones and Singleton analyzed the nitration of toluene in dichloromethane at the M06-2X/6-311G(d) level by different computational approaches [7]. They found that the experimental regioselectivity could not be reproduced by an analysis of the stationary points obtained by an implicit solvent model, i.e., PCM. The reaction was further studied by a potential mean force (PMF) analysis obtained with umbrella sampling MD simulations and explicit consideration of the solvent (AM1). The PMF curve for the attack of NO_2_^+^ toward the aromatic was found to be downhill and lacked a barrier for the C-N bond formation. Thus, the stationary points obtained by the implicit solvent model were found to have no counterparts with the explicit solvent consideration. However, the PMF analysis predicted 95% para substitution and performed worse than the implicit solvent model. The authors were finally able to reproduce regioselectivity by analyzing the trajectories of unconstrained MD simulations with explicit consideration of the solvent and the counterion.

The results of Nieves-Quinones and Singleton are discouraging as they suggest that predictions of positional selectivity for the nitration of arenes require long DFT-MD simulations with explicit representation of counterion and solvent. However, it is not obvious that their observations are directly transferable to other arenes and to more polar solvents. It should be noted that the methyl substituent is weakly activating, and stronger activating or deactivating substituents may significantly alter the potential energy surface. Furthermore, the counterion effects observed by Nieves-Ouinones and Singleton may not be present in a more polar solvent, and solvents of higher polarity are likely to induce a barrier for the addition of NO_2_^+^.

To further elucidate the solvation effects of this reaction and to investigate quantitative models for prediction of regioselectivity are the objectives of the current study. We have employed M06-2X computations with and without PCM to investigate the difference in structure of the stationary points, and their free energies, for the PES of nitration of benzene in the gas phase and in aqueous solution. We have further analyzed the free energies of the relevant stationary points for the nitration of chlorobenzene and phenol in aqueous solution to study the effects of deactivating and activating substituents on the reactivity profile. These studies suggest that the stationary points play a significant role in determining positional selectivity and that a PCM representation of the solvent without consideration of the counterion is sufficient in many cases. This is confirmed by good quantitative predictions of the positional selectivity based on the free energy of the rate-determining transition state for a wider range of substituents. Estimates of the positional selectivity based on the σ-complex energy are confirmed to fail for nitrations while working well for halogenations. These results are rationalized by Hammond’s postulate and the differences in structure between the rate-determining transition state and the σ-complex.

## Computational details

The quantum chemical calculations were performed using the Gaussian09 program suit [20]. All structures were fully optimized using KS-DFT, employing the hybrid functional M06-2X and the integral equation formalism of the PCM solvent model (IEFPCM) with water as solvent. The Pople type 6-311G(d,p) basis set was used for all geometry optimizations, unless otherwise stated. For some of the activated structures we reoptimized the geometries to allow the electronic structure to attain some singlet diradical character, which is expected for a SET mechanism. This was achieved via the use of an unrestricted KS-DFT computation and by mixing the HOMO and LUMO orbitals in the initial orbital guess throughout a geometry optimization. All structures have been characterized by frequency calculations as either a minimum (no imaginary vibration mode) or a TS (one imaginary vibration mode). The TS structures were further characterized by means of IRC calculations, to verify that a particular TS structure indeed connects to the correct energy minima.

In order to evaluate the effects of larger basis sets we performed single point calculations with the 6-311G(2df,2p) basis set. Whereas this overall had minor effects on the relative energies, generally less than 1 kcal mol^-1^, it sometimes lead to an unphysical change in the energy difference between two neighboring stationary points, e.g., a transition state became lower or equal in free energy to the preceding intermediate (see Supporting information for details). This is most likely an effect of the potential energy surface being very flat in extended regions.

All energies are reported as standard Gibbs free energies, if not otherwise stated. The thermal corrections to Gibbs free energies (Δ*G*) were obtained from frequency calculations employing the harmonic oscillator, rigid rotor, and ideal gas approximations. We further assumed a temperature of 298.15 K and a standard state concentration of 1.0 M. Since the program’s default is 0.0408 M (i.e., 1 atm in gas phase) we have adjusted the reported free energies in the reactions where the number of molecules changes in order to correct for the concentration differences. The free energy correction *ΔΔG*_*react*_ is, e.g., -1.9 kcal mol^-1^ for complexation of NO_2_^+^ with an arene, according to Eq. (1).

1 $$ \varDelta \varDelta {G}_{react}=\varDelta {G}_{react}\left(1\mathrm{M}\right)-\varDelta {G}_{react}\left(0.0408\mathrm{M}\right)=-1.9\;\mathrm{kcal}/\mathrm{mol} $$

Symmetry corrections to the Gibbs free energy, ΔG_sym_, were included via

2 $$ \Delta {G}_{sym}=- RTln\left(\frac{\prod {\sigma}_p}{\prod {\sigma}_r}\right) $$

where σ_p_ and σ_r_ are the symmetry numbers of the products and reactants, as described in references [21, 22]. For the substituted benzenes, the ortho and meta sites are twofold degenerate with respect to the para site. To compensate for the degeneracy, the ΔG of the ortho and meta substitutions are corrected by a factor -*RT*ln(2) = −0.41 kcal mol^-1^.

The majority of the recent computational chemistry studies dealing with aromatic nitration have used KS-DFT and the popular B3LYP functional [5, 12, 14, 18, 23], but we found it appropriate to use the M06-2X functional. This is a non-local hybrid meta exchange-correlation functional that has been parametrized for non-metals and optimized against broad and diverse databases [24]. It is particularly recommended for applications involving main-group thermochemistry (e.g., ionizing potential and π-systems), kinetics (barrier heights), and non-covalent interactions — all areas where it performs strikingly better than B3LYP [24].

In this context, it should be emphasized that B3LYP severely overestimates the driving force for SET between NO_2_^+^ and benzene in the gas phase. This reaction is exothermic by 21.8 kcal mol^-1^ at the B3LYP/6–311++G(d,p) level, whereas the experimental value is 7.4 kcal mol^-1^ [25]. M06-2X also overestimates the exothermicity but to a much smaller extent, at the M06-2X/6-311G(2df,2p) level the SET is exothermic by 13 kcal mol^-1^. Thus, M06-2X seems to be better suited than B3LYP for studying S_E_Ar nitrations. Nieves-Quinones and Singleton also reached the conclusion that M06-2X is well suited for the nitration of arenes [7], and they used a basis set (6-311G(d)) almost identical to that used in the current study.

## Results and discussion

We have chosen to analyze the stationary points on the potential energy surface (PES) for nitration of benzene, one deactivated monosubstituted benzene, chlorobenzene, and one activated, phenol. This selection of aromatic substrates has made it possible to characterize the full PES, including the deprotonation step, of nitration in aqueous solution.

### Benzene

**Fig. 2:**
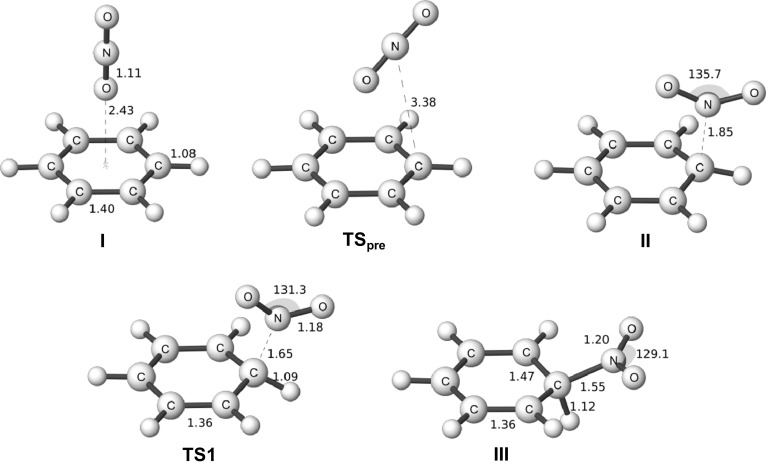
Structures of stationary points in the gas phase nitration of benzene optimized at the M06-2X/6-311G(d,p) level. Bond lengths in Angstroms and angles in degrees. Adapted from [6] with permission from John Wiley & Sons, Inc., Copyright

**Fig. 3:**
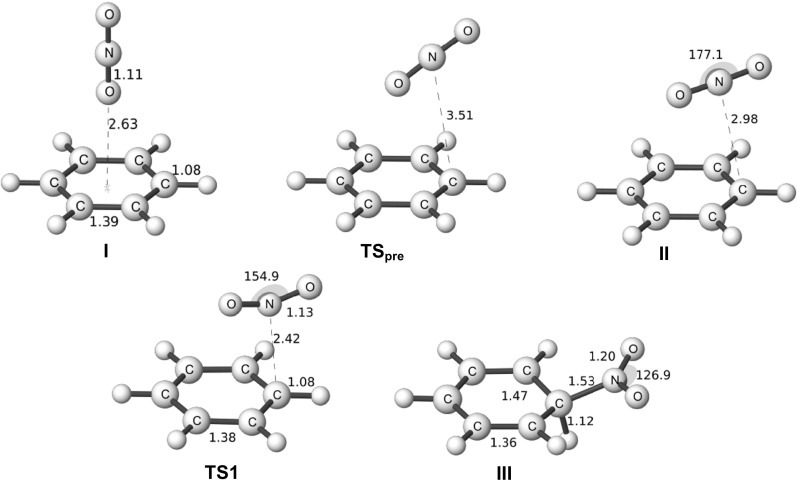
Structures of stationary points in the nitration of benzene in aqueous solution optimized at the M06-2X/6-311G(d,p) level. Bond lengths in Angstroms and angles in degrees. Adapted from [6] with permission from John Wiley & Sons, Inc., Copyright

We will begin our analysis with the nitration of benzene, where we have studied both the gas phase and solution reaction. The stationary points have previously been reported in a book chapter [6]. Between the reactants and the σ-complex (III), we have found two intermediates on the gas phase PES. These correspond to the ones found by Esteves et al. [5]. The first structure (I) is the unoriented π-complex with the linear nitronium ion oriented perpendicularly above the ring plane. The second (II) is an oriented reaction complex, which has the nitrogen of the nitronium ion coordinated directly to one of the ring carbons. We have also characterized the transition state that connects (I) and (II) (TS_pre_), and the transition state leading to (III) (TS1). The stationary points in gas phase and in aqueous solution are of similar types, but their detailed geometries are different as shown in Figs. 2 and 3 [6]. The coordinated π-complex (II) has a much shorter C-N distance in gas phase indicating a strong interaction and a significant degree of charge transfer, while the corresponding structure in solution can be viewed as a weak cation-molecule complex trapped in a solvent cage. The TS1 structure also has a much shorter C-N distance in gas phase than in aqueous solution, while the σ-complex structures are very similar. These structural differences indicate that the TS1 comes much earlier along the reaction coordinate in solution than in the gas phase, and this can be explained by the significant cost in solvation energy of going from a system in II with the charge localized at the NO_2_ group to the charge delocalized system in TS1.

**Fig. 4:**
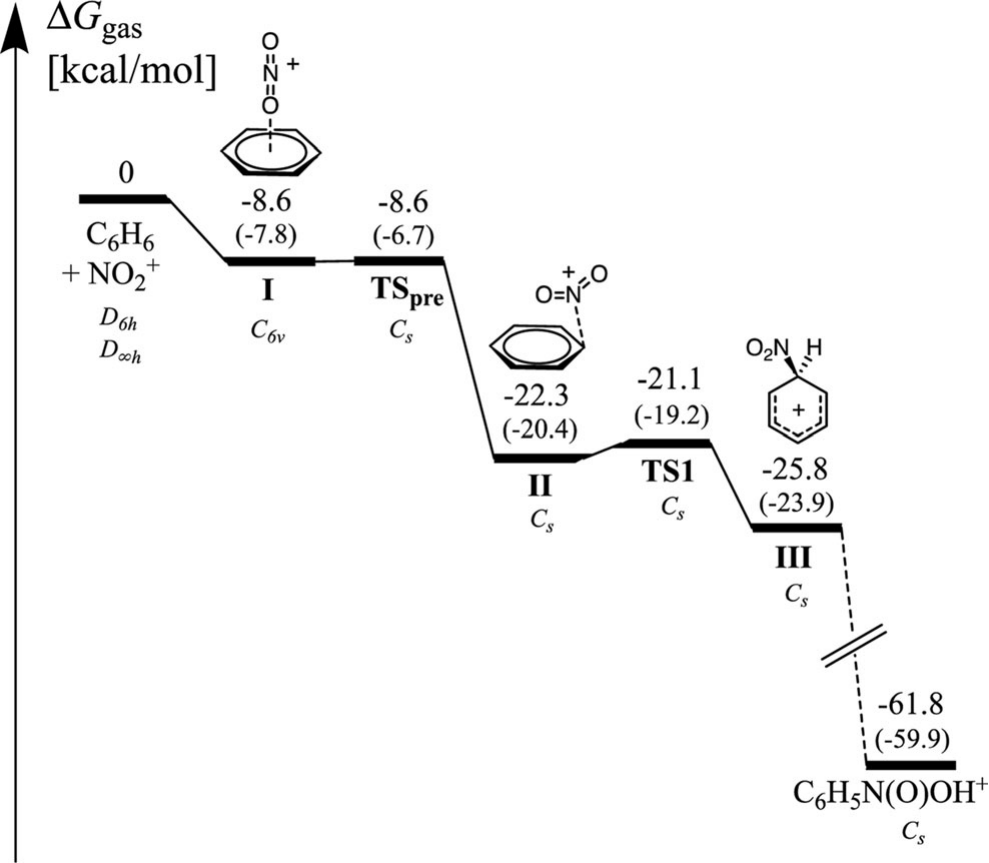
The free energies of the stationary points on the PES for the gas phase nitration of benzene computed at the M06-2X/6-311G(d,p) level. Included in italics is the corresponding point group symmetry at the different stationary points. Free energies without symmetry corrections are given in parentheses

**Fig. 5:**
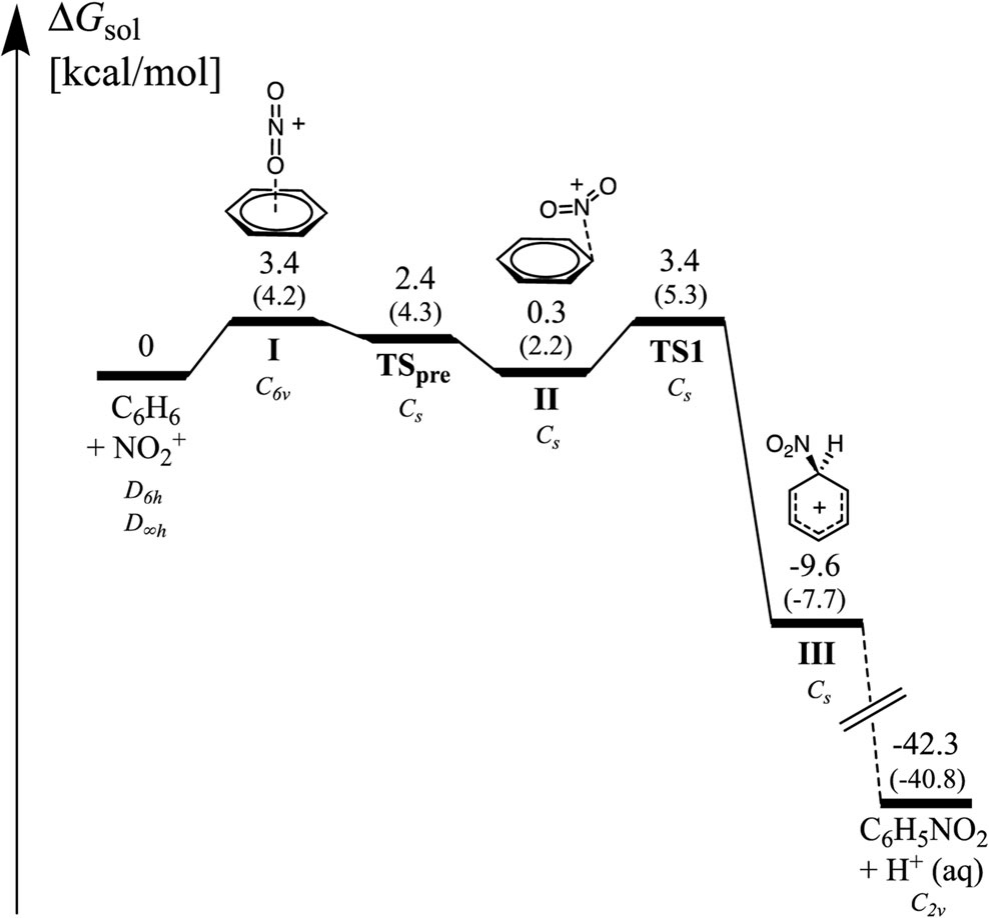
The free energies of the stationary points on the PES for the nitration of benzene in aqueous solution computed at the M06-2X/6-311G(d,p) level. Included in italics is the corresponding point group symmetry at the different stationary points. Free energies without symmetry corrections are given in parentheses

The overall free energy profile for nitration in gas phase is indeed very different from that in solution, as shown in Figs. 4 and 5. In gas phase, the formation of the σ-complex from the free reactants is an exergonic process with a zero overall barrier. In aqueous solution, the formation of the first π-complex (I) is an endergonic process by 3.4 kcal mol^-1^. The transition state (TS_pre_) for the conversion into (II) is slightly lower in free energy than the intermediate (I), and most likely the barrier for conversion into (II) is very low. The reason for the lower free energy of TS_pre_ compared to I is the higher symmetry of the latter, i.e., after forming I the system can continue to pass a TS_pre_ at any of the six carbons. Without the symmetry correction TS_pre_ is higher in free energy than I by 0.1 kcal mol^-1^. However due to the very flat potential energy surface of benzene, which is similar to toluene, the relevance of I and TS_pre_ for the kinetics of the reaction is uncertain. The intermediate (II) has a free energy that is 3.1 kcal mol^-1^ lower than (I). Our free energy corrected PES indicates that TS1 is similar in energy to I and TS_pre_. However, since the reaction channel is expected to be narrower around this location, TS1 is likely to be the rate-determining transition state. After TS1, the free energy drops by 13 kcal mol^-1^ to form the σ-complex (III).

### Chlorobenzene

**Fig. 6:**
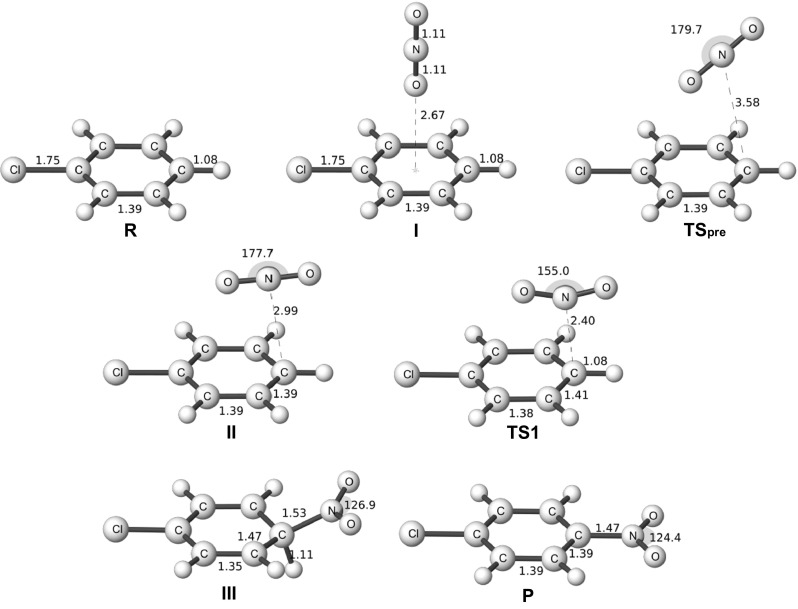
The structure of the para isomer for the stationary points on the PES for nitration of chlorobenzene. Bond lengths in Angstroms and angles in degrees

The monosubstituted chlorobenzene is deactivated in S_E_Ar and the substituent is known to have an ortho/para directing effect. Focusing only on the reaction in solution, the initial part of the PES is similar to benzene both in the structure and the free energy of the stationary points. The structure of the para isomer for all stationary points on this PES up to the formation of the σ-complex together with the final product are depicted in Fig. 6. The standard free energies for forming the ortho, meta, and para isomers are given in Table 1, together with the corresponding energies for benzene and phenol. For chlorobenzene, TS1 is significantly higher in free energy than for benzene, and is indicated to be rate-determining in accordance with the generally accepted picture for this reaction. Furthermore, the free energies of the different TS1 isomers reproduce the experimentally observed ortho/para directing effect. However, the computations predict a slight preference for the para position by 0.3 kcal mol^-1^, whereas according to experiment the ortho position is slightly preferred by 0.5 kcal mol^-1^ (Table 3). The σ-complex is a poor model for TS1, and predicts a nearly 100% formation of the para-isomer with the ortho and meta isomers lying more than 5 kcal mol^-1^ higher in energy.

**Table 1:** Free energy differences from reactants for the nitration of benzene, chlorobenzene, and phenol in solvent

Species	Δ*G* energy difference from reactants (kcal mol^-1^)		
	Benzene	PhCl	PhOH
Reactants (NO_2_^+^ + benzene/PhCl/PhOH) (symmetry-corrected)	0.0	0.0	0.0
π-complex **(I)**	3.4	3.4	2.5
TS_pre_, ortho	2.4^a^	4.2	2.8
TS_pre_, meta	–	4.0	3.1
TS_pre_, para	–	4.3	3.4
reaction complex **(II)**, ortho	0.3^a^	3.3	1.8
reaction complex **(II)**, meta	–	2.7	1.9
reaction complex **(II)**, para	.	1.9	1.1
TS1, ortho	3.4^a^	7.0	2.6
TS1, meta	–	8.2	5.3
TS1, para	–	6.7	1.8
σ-complex **(III)**, ortho	−9.6^a^	−2.6	−18.8
σ-complex **(III)**, meta	–	−0.6	−6.8
σ-complex **(III)**, para	–	−7.4	−25.1
σ-complex **(III)**_w_, ortho	–	–	−18.7 ^b^
σ-complex **(III)**_w_, para	–	–	−25.1^b^
TS2_w_, ortho	–	–	−17.6^b^
TS2_w_, para	–	–	−23.7^b^
Product, ortho	−42.3^a,c^	−33.8^c^	−42.7^c^
Product, meta	–	−40.0^c^	−41.8^c^
Product, para	–	−40.2^c^	−43.2^c^

^a^Obviously, it is not the ortho position for benzene as all positions in benzene are degenerate

^b^With one water molecule associated with the species

^c^PhNO_2_ + H^+^(aq), PhClNO_2_ + H^+^(aq) and PhOHNO_2_ + H^+^(aq)

### Phenol

**Fig. 7:**
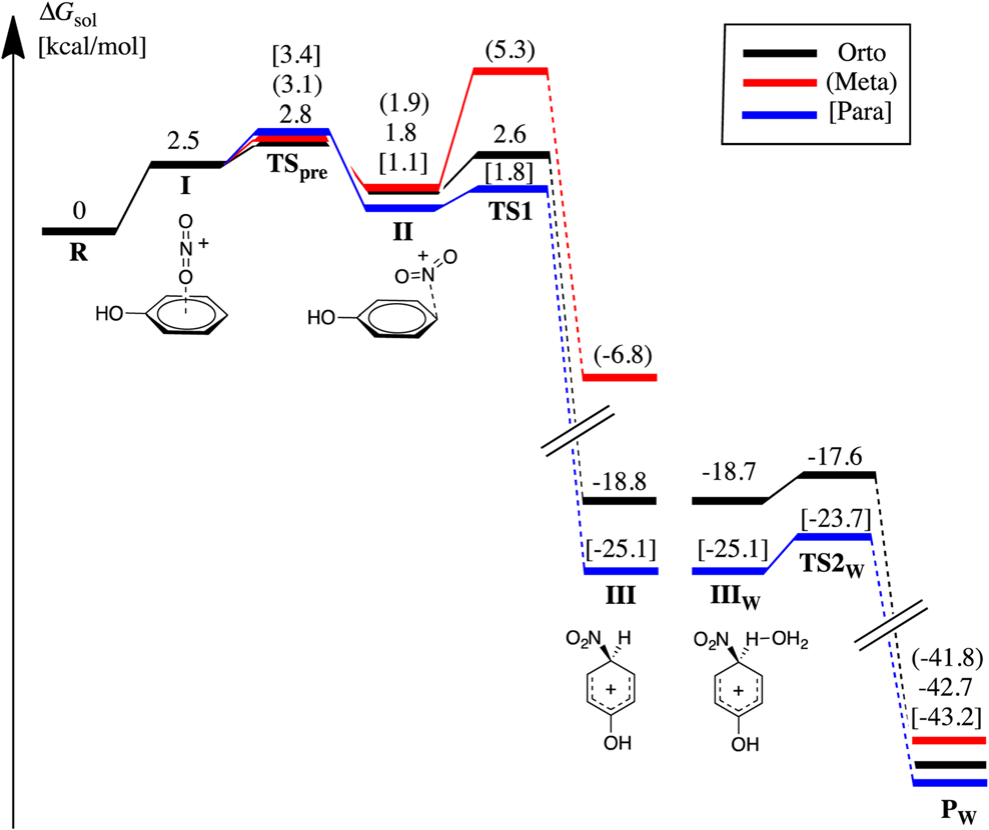
Standard free energies at the stationary points on the PES for the nitration of phenol in aqueous solution (1 M, 298.15 K). The species to the left of the gap are calculated with the bare PCM description. The three species to the right, indexed with “w” as in water, are calculated with PCM and one explicit water molecule coordinated to the structures. The energy for the (III_w_) para isomer has been leveled with the corresponding para structure (III). Note that a *C*_2v_ point group symmetry was assumed for phenol (R) due to the near degeneracy of the ortho and meta sites with H directed toward or away from the site. The difference between the ortho TS1 for the different sites is, e.g., 0.16 kcal mol^-1^

Phenol is activated compared to benzene and ortho/para directing. For phenol we have found all stationary points, including TS2 (the expulsion of the proton) for the ortho and para isomers (vide infra). The whole PES for the nitration of phenol is shown in Fig. 7. In contrast to chlorobenzene, TS1 is the rate determining step only for the meta isomer. For the para isomer the formation of the reaction-complex (II) is rate determining and lies 1.6 kcal mol^-1^ above TS1 in free energy. For the ortho isomer the two transition states TS_pre_ and TS1 are very close in free energy. Considering TS_pre_ as rate-determining for ortho- and para-substitution and TS1 as rate-determining for meta-substititution leads to an almost perfect agreement with the experimental positional selectivity; the relative activation free energies for the ortho, meta, and para positions are 0, 2.5, and 0.3 kcal mol^-1^ according to theory and the corresponding experimental estimates are 0, 1.8, and 0.3 kcal mol^-1^.

In order to evaluate to what extent the phenol nitration reaction follows a SET type mechanism, in line with the hypothesis of Esteves and coworkers [5], we optimized all of the stationary points on the PES up to the σ-complex with unrestricted M06-2X computations, and investigated the singlet diradical character. In all cases we found that the Kohn-Sham wavefunction converged toward a restricted solution, as shown by the zero expectation value of the spin operator (<S^2^>). Even though there appears to be a consensus that some degree of SET is present in nitrations and that this is most prominent for activated aromatics, it seems to be relevant only for gas phase conditions. This is not surprising considering that the solution reaction lacks the driving force for SET, and we conclude that the polar (Ingold-Hughes) mechanism dominates here.

In order to find TS2 for the ortho and para isomers, we had to take explicit solvent effects into consideration. To this end, we used one explicit water molecule and optimized the TS2 structures with PCM. In this reaction step the proton is transferred to the water molecule, and forms a H_3_O^+^ molecule that subsequently can leave the system. The position of the explicit water molecule is unambiguous for the TS structure, but less stringent for the preceding σ-complex or for the proceeding product. In order to identify the relevant structures we performed an IRC calculation in both directions from the TS2 to find the closest minima.

**Fig. 8:**
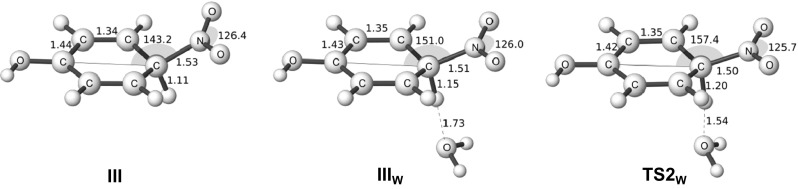
Some para isomer structures for the nitration of phenol. The σ-complex, with and without an explicit water molecule, and the TS2, the expulsion of the proton. Bond lengths in Angstroms and angles in degrees

The difference in free energy between the ortho- and para-positions for the σ-complex with one explicit water molecule is almost identical to the corresponding energy difference for the σ-complexes without explicit water. The free energy barriers going from the ortho- and para σ-complex structures to the corresponding TS2 are only 1.1 and 1.4 kcal mol^-1^, respectively. We have not found any experimentally based determinations of the energy barriers for this last step, nor any relevant computational estimates, but our values agree well with the common observation that this step is very fast and not rate determining [1, 8, 9]. The structures of TS2 and the preceding σ-complex are shown in Fig. 8. Our results indicate that even a weak base, such as water, can act as a base and deprotonate the σ-complex in a nearly barrierless process, making the deprotonation a much faster process than the formation of the σ-complex. We, furthermore, note that the activated S_E_Ar reaction (for instance ortho/para nitration of phenol) seems to have a stabilized σ-complex compared to the deactivated cases (e.g., meta nitration of phenol or nitration of chlorobenzene). The higher stabilization of the σ-complex leads to a barrier for proton transfer, and allows for the characterization of the transition state for this process.

### General observations

The distance between the nitrogen atom of the nitronium electrophile and the para carbon in the aromatic ring and the angle between the nitrogen and the two oxygens of the nitronium ion for benzene, chlorobenzene, and phenol are shown in Table 2. This distance and the angle is continuously decreasing along the potential energy surface. It is also clear from these results that the transition state for forming the σ-complex comes much earlier for phenol. In fact, the rate-determining transition state for the ortho and para isomer of phenol is the formation of the reaction complex (II), while it is the formation of the σ-complex for chlorobenzene.

**Table 2:** Geometric features of the stationary points for the nitration of benzene and of the para position of chlorobenzene and phenol. Distance between the nitrogen of the NO_2_-group and the para-carbon of the ring, O-N-O angle of the NO_2_-group

Species	C4-N distance (Å)			O-N-O angle (°)		
	Benzene	PhCl	PhOH	Benzene	PhCl	PhOH
Reactants (NO_2_^+^ + benzene/PhCl/PhOH)	–	–	–	180.0	180.0	180.0
π-complex **(I)**	3.99	4.08	3.96	180.0	179.9	180.0
**TS**_**pre**_	3.50	3.55	3.50	179.4	179.6	179.4
reaction complex **(II)**	2.98	2.99	2.82	177.1	177.7	173.1
**TS1**	2.42	2.40	2.67	154.9	154.9	167.2
σ-complex **(III)**	1.52	1.53	1.53	126.9	126.9	126.4
σ-complex **(III)**_w_	–	–	1.51	–	–	126.0
TS2_w_	–	–	1.50	–	–	125.7
Product (PhCl)/Product_w_ (PhOH)	1.47	1.47	1.47	123.2	124.4	124.5

In solution, the formation of the first π-complex (I) is an endergonic process by 2.5–3.4 kcal mol^-1^, and there is a significant barrier for forming the σ-complex. The rate-determining transition state for nitration in solution is early and shows very little resemblance to the σ-complex. The energy of this transition state is also much closer to that of the reaction complex (II) than the σ-complex, which is in good agreement with the early proposal of Olah. He reached a number of conclusions regarding the nature of the transition states in S_E_Ar reactions, based on the experimental stability data for π- and σ-complexes of arenes and the low substrate but high positional selectivity in highly exothermic S_E_Ar reactions [2]. Olah suggested that the rate-determining transition state is the one leading to the reaction complexes (II), which should be followed by a transition state that differs substantially between the isomers and leads to the σ-complex. In contrast for S_E_Ar reactions that are deactivated, either by means of a less nucleophilic aromatic substrate or a weaker electrophile, the rate-determining step was proposed to be the formation of the σ-complexes [2].

It is striking how well Olah’s conclusions of both the activated case and the deactivated case agree with the free energies of the stationary point on the PES for phenol and chlorobenzene, respectively. Olah also noted that, in view of experimental observations regarding substitution rates and π-complex and σ-complex stabilities, “the wide acceptance of the view that transition states of electrophilic aromatic substitutions generally resemble σ-complexes is surprising” [2]. Our calculations also agree with the proposal that the TS resembles the π-complex for activated cases. Thus, the conclusions reached by Olah over 40 years ago based on experimental data are supported by the quantum chemical calculations reported in this study.

### Positional selectivity

One objective of the present study has been to investigate the stationary points on the PES to evaluate what precision can be obtained in the prediction of positional selectivity. To this end we located TS1 and the σ-complex (III) for all the isomers of five monosubstituted benzenes, PhX, (X = CN, CHO, Br, Cl, and OH) in condensed phase and compared the results with experimentally reported isomer distributions. The experimental isomer ratios have been recalculated to free energy differences, where we have used the actual experimental reaction temperature in the calculations. The accuracy of this approach is evaluated in terms of how well the standard Gibbs free energy (ΔG) values correspond to the same values obtained from experiments and we quantified it as the mean absolute deviation (MAD) for each arene. In order to measure the accuracy, we proceeded in the following way: We formed the ∆E or ∆G differences between all three combinations of isomers, took the differences between the experimental and calculated results for all three combinations and then formed an absolute average.

The level of theory used in the current study is sufficient to give a good quantitative reproduction of the regioisomer distribution. The MAD between TS1 and experimental values for Δ*G* lies in the range 0.1–1.1 kcal mol^-1^ and seen as an average of these five reactions it is 0.7 kcal mol^-1^. It is interesting, but certainly not surprising, to note that it is essential to take solvent effects into account in order to obtain results with this accuracy. The results are presented in Table 3. If we take into consideration that for phenol different transition states are rate determining for different isomers, the MAD for the predicted isomer distribution of this system improves from 1.3 kcal mol^-1^ to 0.4 kcal mol^-1^.

**Table 3:** Free energy differences Δ*G* between isomers in comparison to experimental data. Corresponding values for (Δ*E*) given in parenthesis. All values are given in kcal mol^-1^

PhX; X=	isomer	TS1	TS1^a^ in gas phase	σ-complex (III)	Exp.	MAD (TS1) Solvent/In gas phase
CN	Ortho	0.9 (0.6)	0.0 (0.1)	1.9 (1.7)	0.9^b^	0.1 (0.3)/2.4 (1.6)
CN	Meta	0.0 (0.0)	2.1 (1.0)	0.0 (0.0)	0.0^b^	
CN	Para	1.6 (0.9)	0.3 (0.0)	0.8 (0.9)	1.8^b^	
CHO	Ortho	0.2 (0.1)	0.0 (0.0)	3.0 (3.0)	0.4^b^	0.1 (0.3)/1.7 (1.9)
CHO	Meta	0.0 (0.0)	2.2 (2.5)	0.0 (0.0)	0.0^b^	
CHO	Para	1.8 (1.0)	2.6 (1.8)	2.5 (2.5)	1.9^b^	
Br	Ortho	0.1 (0.3)	0.0 (0.0)	4.5 (4.7)	0.0^c^	1.1 (1.4)/1.9 (2.1)
Br	Meta	1.3 (1.0)	1.9 (1.7)	6.3 (6.8)	2.8^c^	
Br	Para	0.0 (0.0)	2.7 (2.4)	0.0 (0.0)	0.4^c^	
Cl	Ortho	0.3 (0.3)	0.0 (0.0)	4.9 (4.9)	0.0^c^	1.1 (1.4)/2.2 (1.9)
Cl	Meta	1.6 (1.2)	1.9 (2.1)	6.8 (7.2)	3.0^c^	
Cl	Para	0.0 (0.0)	2.7 (2.1)	0.0 (0.0)	0.5^c^	
OH	Ortho	0.8, *0.0* ^*e*^ (0.8)	5.5 (4.6)	6.3 (6.5)	0.0^d^	*0.4* ^e^ (*0.7*)^e^/4.9 (4.3)
OH	Meta	3.5, *2.5* ^*e*^ (3.2)	0.0 (0.0)	18.3 (19.4)	1.8^d^	
OH	Para	0.0, *0.3* ^*e*^ (0.0)	2.2 (1.5)	0.0 (0.0)	0.3^d^	
Average						0.7 (0.9)/2.6 (2.4)

^a^Single point gas phase calculations at the PCM-optimized structures

^b^HNO_3_ in TFA at 25 °C [26]

^c^HNO_3_ and H_2_SO_4_ at 45 °C 2 h [27]

^d^10% HNO_3_ in Ac_2_O at 10 °C [28]

^e^If consideration is taken for both TS1 and TS_pre_

Several authors have argued that the σ-complex (III) is a good model of the rate-determining transition state [17–19]. In this study the relative energies of the isomers of this intermediate provide a correct qualitative ranking of the meta isomer as the major (X = CN, CHO) or minor (X = Br, Cl, OH) product. However, the energy differences between isomers for (III) are far larger than the ones corresponding to the experimentally found isomer distribution, giving an accuracy for the five investigated monosubstituted benzenes, measured as MAD, which ranges from 1.1 to 11.2 kcal mol^-1^. This is essentially the same picture as we obtained in our previous study [17].

It is also of interest to investigate if the reaction complex (II) is a better model of that same transition state. According to our calculations of the PES of benzene, chlorobenzene, and phenol, this reaction intermediate is closer to the rate-determining transition state than the σ-complex, both in geometry and in energy. However, the energy differences between the isomers of (II) are rather small and they do not provide even a qualitatively correct reproduction of the isomer distribution. More specifically, the energy of the meta isomer of (II) is too low relative to the energies of the ortho and para isomers, with the result that the amount of meta isomer is overestimated. The MAD between the energies of the reaction complexes (II) and experimental values is 2.0 kcal mol^-1^ for chlorobenzene and 1.2 kcal mol^-1^ for phenol. This appears to be better than for (III), but this is more an effect of the energy differences being smaller for the isomer of (II) than for (III). Thus, if the objective of the modeling is to obtain positional selectivity predictions of some accuracy, neither the reaction complex (II) nor the σ-complex (III) are suitable as models of the rate-determining transition state for S_E_Ar nitrations.

### Global reactivity

We also calculated the energy barrier from reactants to the rate-determining transition state for the most abundant isomer as well as for the corresponding σ-complex (III) for the six investigated benzenes. These results are shown in Table 4 and correspond well to the reactivity ranking according to the textbook, where X = H is the reference, X = OH is put as “strongly activating”, X = Cl or Br to “weakly deactivating”, X = CHO to “moderately deactivating” and X = CN to “strongly deactivating” [29]. Unfortunately, we have not been able to carry out a quantitative correlation of the global reactivity, due to the scarcity of experimental kinetic data run under comparable reaction conditions. An earlier attempt to correlate experimental rate constant with substituent constants using Hammet plots found a better correlation with σ^+^_m_ /σ^+^_p_ than with σ_m_ /σ_p_ [30]. This was taken as an indication of a rate-determining transition state where the substituent has a direct resonance interaction with a positive charge. However, it should be noted that the data set contained only weekly activating and deactivating substituents, and thus there were only minor differences between the σ^+^_m_ /σ^+^_p_ and σ_m_ /σ_p_ values. Our computed activation free energies (without symmetry and degeneracy corrections) correlate only weakly with σ_m_ /σ_p_ (*R* ^2^ = 0.87), and the correlation is even weaker with σ^+^_m_ /σ^+^_p_ (*R* ^2^ = 0.69). The most activated system, phenol, is the outlier in these correlations, and *R* ^2^ improves to 0.97 for both types of substituent constants when this compound is removed. The correlations are consistent with the early rate-determining TS (TS_pre_ or TS1), where the positive charge resides entirely on the NO_2_ moiety and no bond has yet been formed between the nitrogen and the aromatic carbon. In contrast the free energy of σ-complex formation correlates much better with σ^+^_m_ /σ^+^_p_ (*R* ^2^ = 0.97) than with σ_m_ /σ_p_ (*R* ^2^ = 0.95). This is not surprising considering that there is potential for a direct resonance interaction between the substituent and the positive charge at the substitution site in the σ-complex. In line with these observations, we note that there is a much larger substituent effect on the free energy for σ-complex formation compared to the activation free energy; the former varies by almost 30 kcal mol^-1^, whereas the latter only has a span of 8.7 kcal mol^-1^ for our data set.

**Table 4:** The difference in free energy between the rate-determining transition state and σ-complex of the main isomer and the reactants (kcal mol^-1^). Substituent constants from ref. [30]

PhX; X=	ΔG^a^ (TS– [PhX + NO_2_^+^])	ΔG^a^ (σ-complex– [PhX + NO_2_^+^])	σ_m_ /σ_p_	σ^+^_m_ /σ^+^_p_
CN (m)	11.7^b^ (12.9)^c^	4.1 (5.3)^c^	0.56	0.56
CHO (m)	7.9^b^ (9.1)^c^	−2.2 (−1.0)^c^	0.35	0.35
Br (p)	6.8^b^ (7.6)^c^	−7.1 (−6.3)^c^	0.23	0.15
Cl (p)	6.7^b^ (7.5)^c^	−7.4 (−6.6)^c^	0.23	0.19
H	3.4^b^ (5.3)^c^	−9.6 (−7.7)^c^	0	0
OH (p)	3.4^d^ (4.2)^c^	−25.1 (−24.3)^c^	−0.37	−0.92

^a^adjusted for symmetry and site degeneracy

^b^ΔG (TS1–[PhX + NO_2_^+^]) were used as the rate-determining transition state for these cases

^c^ΔG without symmetry or site degeneracy corrections

^d^ΔG(TSpre-[PhX + NO_2_^+^]) was used as the rate-determining transition state for this case

The relative stabilities of the final products (Table 1) do not show any correlation with the experimentally determined isomer distribution (Table 3), and thus support our assumption of kinetic control in S_E_Ar nitrations. Furthermore, thermodynamic control is inconsistent with the large exothermicities of the nitrations.

### The σ-complex approach for S_E_Ar halogenations and S_E_Ar nitrations—a comparison

As mentioned in the introduction and in the previous section, there have been several attempts to use the σ-complex approach for reactivity predictions of nitrations [18, 19]. This approach can give good correlations when the energies of σ-complex formation are related to experimental rate constants for different substrates, since the rate constants typically span many orders of magnitude. However, if the purpose is prediction of positional selectivity the approach fails to give even semi-quantitative accuracy. This is in sharp contrast to halogenations, where the σ-complex approach is of sufficient accuracy to be used for quantitative predictions of positional selectivity [17, 19]. The MADs previously obtained by us for monosubstituted benzenes were 2.2 kcal mol^-1^ for nitrations and 0.4 kcal mol^-1^ for chlorinations [17].

What is the reason for this discrepancy? In a previous paper [17] we stated that “the electrophiles involved in halogenations are usually less reactive than the electrophiles used in nitrations and Friedel-Crafts reactions. When the Hammond postulate is applied, this implies that halogenations have a later transition state than nitrations and Friedel-Crafts reactions. Thus, if the Hammond postulate is applicable to S_E_Ar reactions, we would expect halogenations to show a closer structural similarity between the σ-complex and the corresponding transition state for its formation. In this study we will evaluate the validity of this hypothesis.

It is instructive to begin by comparing our five examples of nitration of monosubstituted benzenes. If we take the para isomer as model, the distance between the nitrogen of the NO_2_^+^ group and the para ring carbon for the TS1 increases as the substituent becomes more activating, indicating that the rate determining TS indeed becomes earlier as the reaction rate increases. The earlier the TS1 lies along the reaction coordinate, the lower similarity it has with the σ-complex (III), and thus the worse can we expect the σ-complex approach to perform. Phenol has the earliest TS1 among our investigated cases, and this is the case where the rate determining TS structure is furthest away from the corresponding σ-complex structure both geometrically and energetically. TS_pre_ is the rate-determining transition state for the para isomer in the case of phenol, and this structure is obviously even further away from the corresponding σ-complex (III) than the TS1 structure. Table 5 gives a summary of the distances between the para-carbon and the N-atom (C4-N), the difference in free energy, and how well the σ-complex approach performs in terms of MAD.

**Table 5:** Nitration. C4-N (para-carbon and N) distances, differences in free energy, and MAD for the σ-complex and TS1 for PhX-NO_2_

PhX; X=	Distance TS1 [Å]	Distance σ-complex [Å]	Distance difference^a^ [Å]	ΔG (TS1-σ-complex) [kcal mol^-1^]	MAD TS1 [kcal mol^-1^]	MAD σ-complex [kcal mol^-1^]
CN	1.934	1.530	0.404	8.0	0.1	1.1
CHO	2.129	1.527	0.602	9.3	0.1	1.7
Br	2.393	1.529	0.864	13.9	1.1	3.3
Cl	2.397	1.529	0.868	14.2	1.1	3.5
OH	2.673	1.530	1.143	26.8	0.4^b^	11.2
OH^c^	3.504	1.530	1.968	28.5	0.4^b^	11.2

^a^The difference in C4-N distance between TS1 and the σ-complex

^b^If consideration is taken for both TS1 and TS_pre_

^c^TS_pre_

Next we can perform the same investigation of the corresponding chlorinations with Cl_2_; the picture for chlorination is distinctly different from that of nitrations, Table 6 summarizes the distances between the para-carbon and the Cl-atom and the differences in energy. It is obvious that the rate determining TS resembles the σ-complex more closely than for nitrations and also that they are closer in energy.

**Table 6:** Chlorination. C4-Cl (para-carbon and Cl) distances and differences in free energy energies for PhX-Cl_2_ [M06-2X/6–311+G(d,p)]

PhX; X=	Distance TS1 [Å]	Distance σ-complex [Å]	Distance difference^a^ [Å]	ΔG (TS1-σ-complex) [kcal mol^-1^]
CN	1.993	1.779	0.214	0.8
CHO	1.938	1.777	0.161	1.5
Br	2.009	1.805	0.204	3.6
Cl	2.012	1.797	0.215	3.6
OH	2.162	1.808	1.354	10.5

^a^the difference in C4-Cl distance between TS1 and the σ-complex

**Fig. 9:**
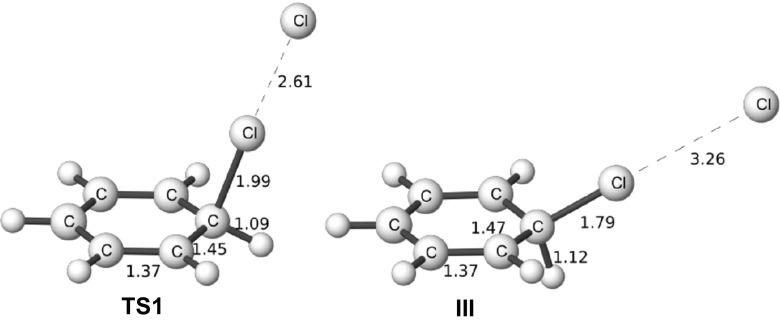
Para isomers for the TS1 and σ-complex structures (III) for the chlorination of benzene

The structures of the TS1 and the σ-complex para isomers for the chlorination of benzene are depicted in Fig. 9. These structures can be compared to the corresponding nitration structures in Fig. 3; note the difference in the positioning of the electrophile. In the σ-complex the Cl-Cl distance is increased to 3.3 Å and the structure can essentially be viewed as an arenium ion with a Cl^−^ coordinated to the Cl substituent.

The fact that the σ-complex is significantly different both in energy and in geometry to the rate-determining TS provides a rationale for the failure of the σ-complex approach for S_E_Ar nitrations. Correspondingly, the geometric and energetic similarities between these species for halogenations can explain the success of the σ-complex approach for this reaction type.

In an earlier article concerning *nucleophilic* aromatic substitution reactions (with fluorine as the leaving group) we have also identified the geometric and energetic resemblance between the σ-complex reaction intermediate and the rate-determining transition state as the reason for the excellent correlations between the energies for forming the σ-complex and the experimentally found reaction rate (measured both as correlation coefficient and as MAD) [31]. Thus, both S_N_Ar as well as S_E_Ar nitrations and S_E_Ar halogenations constitute instructive examples if we want to formulate a more efficient approach to investigate whether a simplified reactivity model, based on the energy of the highest reaction intermediate, is likely to be a useful approximation. A suitable approach seems to be first to investigate the full potential energy surface with solvent effects included and compare the geometry and the energy of the rate-determining transition state with the chosen intermediate. If they are too far apart, then the basic assumption of the reaction intermediate approach will be invalid and it is unlikely that such a reactivity model will produce quantitatively reliable results. However, an intermediate that is close in energy or structure to the rate-determining transition state is no guarantee for a useful reactivity model, as was demonstrated by our attempt to use the reaction intermediate (II) as a model for the TS in nitration. Careful validation in each case is certainly required.

## Conclusions

According to our calculations the mechanism of electrophilic aromatic nitrations in solution involves three reaction intermediates, in the following order: first the unoriented π-complex with the linear nitronium ion placed O-down perpendicularly above the ring plane, second the oriented reaction complex with the nitronium ion placed with the N-atom coordinated directly above the respective carbon, and third the classical σ-complex. It is crucial to take solvent effects into consideration both in order to obtain a qualitatively correct picture of the mechanism as well as to obtain quantitatively accurate results for local reactivity and for the relative energy barriers of the different transition states of the reaction. In agreement with Olah’s conclusion from over 40 years ago, we have, furthermore, found that the TS leading to the oriented reaction complex is rate-determining for activated substituents, while the TS leading to the σ-complex is rate-determining in deactivated cases. The last step in the nitration, the expulsion of the proton, could be successfully modeled for the activated phenol compound using one explicit water molecule together with a PCM model. However, no TS for the H^+^ expulsion could be located for benzene or the deactivated chlorobenzene species, indicating a barrierless process in aqueous solution for non-activated arenes.

The simplified reactivity approach using the σ-complex as a model of the rate-determining transition state has previously been shown to be very successful for halogenations but problematic for nitrations. These observations could now be rationalized with the geometric and energetic resemblances (or the lack of resemblances) between the TS1 and σ-complex structures. In the case of nitration, the free energy of the rate-determining transition state gave excellent agreement with experimentally found isomer distributions, but none of the three reaction intermediates could be used to accurately model the positional selectivity.
